# Use Database to Evaluate the Prevalence of Hunger Among Adolescents in Brazil

**DOI:** 10.3389/fnut.2021.773260

**Published:** 2021-11-23

**Authors:** Ana Laura Benevenuto de Amorim, José Raimundo Sousa Ribeiro Junior, Helida Ventura Barbosa Gonçalves, Daniel Henrique Bandoni

**Affiliations:** ^1^Nutrition and Food Service Research Center, Universidade Federal de São Paulo (UNIFESP), São Paulo, Brazil; ^2^Interdisciplinary Program of Health Sciences, Federal University of São Paulo (UNIFESP), Santos, Brazil

**Keywords:** hunger, adolescent, food insecurity, database, food access, nutrition, social determinants of health

## Abstract

Food insecurity and malnutrition have become serious problems in many countries. In recent years, Brazil has experienced an increase in the prevalence of food insecurity and hunger. However, there is limited information on the status of these issues, and food security assessments are only performed as household measures. Therefore, the use of available databases is essential to expand information and support decision-making in the fight against food insecurity. This study aimed to evaluate the relationship between reports of hunger among adolescents and their sociodemographic characteristics. We used data from the 2015 National School Health Survey. The main variable of interest was obtained from responses to the following question: “Over the past 30 days, how often have you gone hungry because you did not have enough food at home?”. The responses were separately gathered from those who reported going hungry and those who did not. Socioeconomic characteristics were evaluated simultaneously. For statistical analysis, a Pearson chi-square test and multiple analyses were performed using Poisson regression models. A total of 101,888 adolescents were evaluated. The variable used to measure hunger was associated with maternal education, internet access, and fruit intake. The results showed a positive association between adolescents who reported going hungry and women, black and indigenous adolescent students living in households with more than five people, adolescents not living with their father, and adolescents planning to work or not knowing what they will do after completing the ninth grade. The results demonstrate that it is possible to use secondary data with a single question to assess, monitor, and provide insights into how food security impacts the sociodemographic groups differently.

## Introduction

Adolescence is the stage of life marked by intense physical, cognitive, and emotional development. Thus, access to a well-balanced diet (one that is sufficient, varied, and complete) is even more relevant during this period (1, 2). Studies have presented the relationship between eating habits and the socioeconomic level of adolescents; youths belonging to more favoured economic classes have unhealthy eating habits, while those from poorer families consume rice and beans more often. During adolescence, nutritional problems that have occurred since childhood can be minimised, and healthy eating habits and lifestyles can be formed (3–7).

Food and Nutritional Security can be defined as a concept that “exists when all people have, at all times, physical, social, and economic access to safe food, consumed in sufficient quantity and quality, that meets their nutritional needs and food preferences in an environment with adequate sanitation and health services, allowing a healthy and active life” (8). There are several methodologies and indicators that can be used to measure food security; however, limited information is available for the assessment of food insecurity (FI) and hunger in specific population groups (9).

Food insecurity and hunger are significant problems in Brazil, and the number of people who experience food insecurity (FI) has increased in recent years, from 37.5 million (18.3%) households in 2013 to 43.1 million (20.6%) households in 2017 and 2018 (10). Data on the status of FI and hunger during the coronavirus disease-2019 (COVID-19) pandemic demonstrate the worsening of this scenario in Brazilian households, with intense growth in the prevalence of hunger (from 5.9 to 9%) and FI (from 36.7 to 55.2%) in the Brazilian population (11).

The prevalence of FI is higher in Brazilian households with children and adolescents than in households without children and adolescents (10). However, limited studies have evaluated the prevalence of hunger among adolescents and their associated factors. Previous studies have shown that FI, especially moderate and severe hunger, in this age group affects physical and emotional development and is related to high rates of school absenteeism and excessive weight (2, 12, 13).

Due to the inter-sectoral, cross-sectional, and multicausal nature of food and nutrition security (FNS), its understanding depends on the association among various aspects of reality. Low income is one of the most important socioeconomic determinants of FI and hunger. Other demographic and socioeconomic factors, such as colour/race, age, household location, access to water and other public utilities, living conditions, number of people per household, level of education, and employment status, are associated with an increased risk of FI (14–17).

Thus, it is fundamental to determine how many adolescents experience hunger. This study aimed to help close this gap and evaluate the relationship between hunger and sociodemographic characteristics among Brazilian adolescents based on the data from the National School Health Survey (PeNSE, 2015).

## Methods

The National Survey of School Health (PeNSE) was first conducted in 2009 through an agreement with the Brazilian Institute of Geography and Statistics and the Ministry of Health with support from the Ministry of Education, and was designed to monitor the risk factors and protect the health and lifestyle behaviours of students in Brazilian schools. This study used data from the third edition of the PeNSE conducted in 2015, which investigated the behavioural risk and health protection factors in a sample of students on their ninth year of primary education in public and private schools throughout Brazil (18). This sample of adolescents adequately represented the youth across Brazil, including all 27 federative units. The survey was approved by the National Commission of Research Ethics (n. 1.006.467), Conep (18).

### Dependent Variable

The key variable of interest was the self-perception of hunger, which was identified based on the following question: “How often during the last 30 days have you gone hungry because you do not have enough food at home?” The response categories were never, rarely, sometimes, most of the time, and always.

For analysis, responses to how often the student was hungry were grouped into the following two categories:

1. Students who reported not going hungry: The responses considered for this category were never and rarely.
2. Students who reported going hungry: The responses considered for this category were sometimes, most of the time, and always.

To evaluate data on the prevalence of hunger obtained by asking a single question, a multiple correspondence analysis was performed to assess the relationship between the major variable of interest and consumption dimension of FI and income proxy. To determine the consumption dimension of FI, the frequency of fruit consumption measure was used, never, 1–3 days in a week, and ≥ 4 days in a week. To measure income proxy, the following variables were assessed:

1. Maternal education, divided into five categories, incomplete primary education (<7 years of education), primary education (8 years of education), secondary education (9 to 11 years of education), higher level of education (>11 years of education), and unknown (due to high level of missing data [24.7%], this category was retained).
2. Internet access at home (yes or no).

### Independent Variables

To evaluate the relationship between self-perceived hunger and sociodemographic factors, the independent variables were grouped into the following categories:

1. Geographic region of Brazil: North, Northeast, Southeast, South, and Midwest.
2. Place of residence: capital or non-capital cities.
3. School area: urban or rural.
4. School administrative status: public or private.
5. Sex: male or female.
6. Race/skin colour: White, Black, Asian, mixed race, or indigenous.
7. Age: 11–13, 14–15, or 16–19 years.
8. Lives with their mother: yes or no.
9. Lives with their father: yes or no.
10. Household size: up to four people, or five or more people.
11. Have any paid work: yes or no.
12. Plans after completing the ninth grade: keep studying fulltime, work fulltime, work and study, others, or do not know.

### Analysis

Data were analysed using descriptive statistics, and Pearson's chi-square test of association was performed to determine the outcome of self-perceived hunger. A *p*-value of < 0.01 was considered significant.

The correlation between hunger (outcome) and sociodemographic characteristics was tested using univariate and multiple Poisson regression models with robust variance, which estimated the crude and adjusted prevalence ratios and their respective 95% confidence intervals (CIs). Statistically significant variables (*p* < 0.01) and those with changes in prevalence ratios of at least 10% were included in the final model. Analyses were performed based on the design of the sample using the statistic package Stata 14.0.

## Results

**Figure 1:**
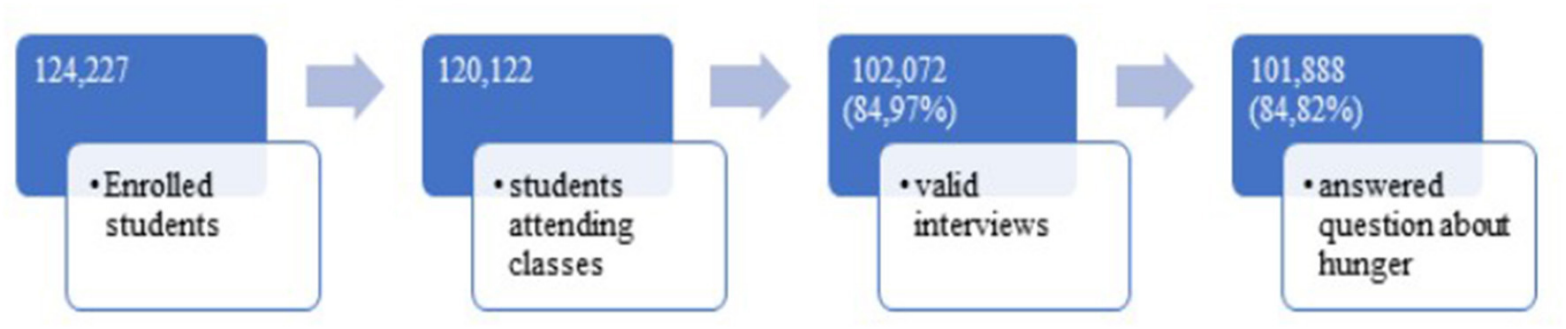
Flowchart of students enrolment in the National Survey of school Health (PeNSE), Brazil, 2015.

Among the 102,072 students interviewed, 101,888 (99.82%) answered the question on how often during the last 30 days they had gone hungry (Figure 1). Among them, 11.18% (*n* = 11,740) confirmed that they had experienced hunger.

**Figure 2:**
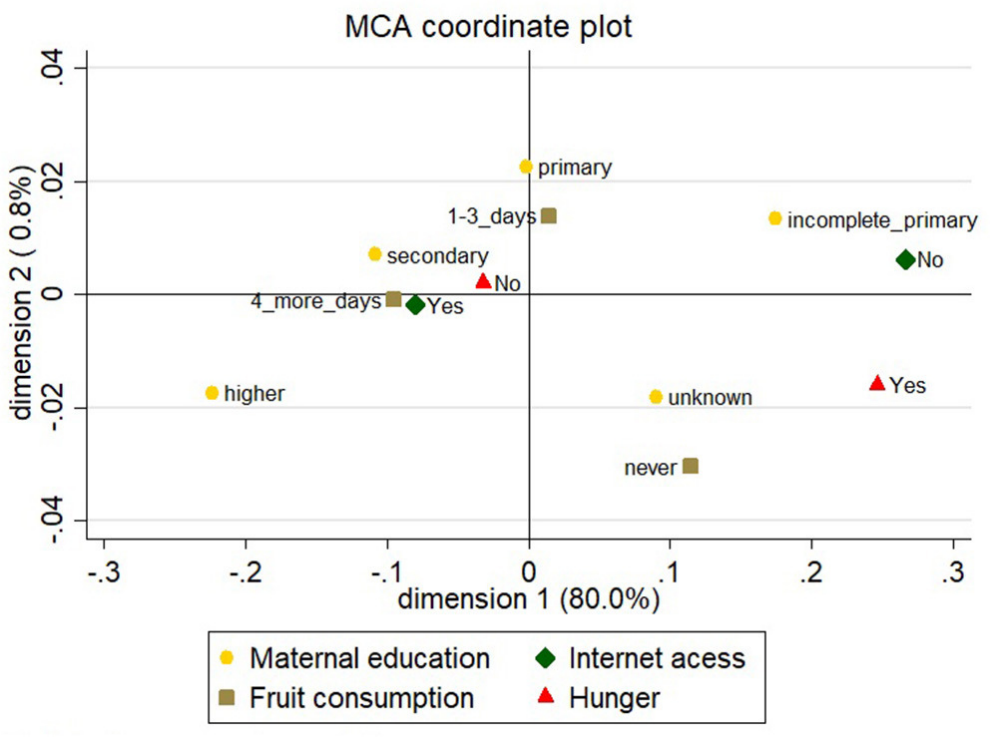
Multiple Correspondence plot.

The multiple correspondence analysis showed that self-perceived hunger was related to poor access to food and lower income levels. The first dimension explained 80% of the variation. We interpreted this dimension to confirm that self-perception of hunger can be used to assess hunger among students (Figure 2).

The following variables were significantly related to the hunger status of the students: geographic region, school administrative status, sex, race/skin colour, age, household size, and plans after completing the ninth grade (Table 1).

**Table 1:** Sociodemographic characteristics according to hunger status, Brazil, 2015.

**Variable**	**Student status**			
	**Not reported hungry**		**Reported hungry**	
	***n***	**(%)**	***n***	**(%)**
Region^*^				
North	20,504	86.40	3,393	13.60
Northeast	32,312	88.45	3,961	11.55
Southeast	16,124	89.67	161	10.33
South	8,753	88.95	1,075	11.05
Midwest	12,455	88.17	1,701	11.83
**Place of residence**				
Capital city	45,301	88.84	5,795	11.16
Non-capital city	44,847	88.81	5,945	11.19
**School area**				
Urban	83,274	89.00	10,356	11.00
Rural	6,874	86.89	1,384	13.11
**School type**				
Public	71,107	88.35	9,877	11.65
Private	19,041	91.58	1,863	8.42
Gender^*^				
Male	43,945	89.92	5,220	10.08
Female	46,203	87.78	6,520	12.22
Race/Colour^*^				
White	30,384	89.91	3,343	10.09
Black	11,126	86.68	1,683	13.32
Yellow	4,014	87.77	560	12.23
Brown	41,382	88.86	5,479	11.14
Indigenous	3,149	86.45	666	13.55
Age (years)^*^				
≤13	15,692	90.70	1,543	9.30
14–15	64,277	89.26	8,075	10.74
≥16	10,179	82.83	2,122	17.17
**Live with the mother**				
Yes	80,115	88.95	10,212	11.05
No	10,005	87.69	1,522	12.31
Live with the father^*^				
Yes	56,653	89.71	6,860	10.29
No	33,421	87.27	4,864	12.73
Household size^*^				
Up to 4 people	52,840	89.97	5,876	10.03
5 or more people	37,292	87.21	5,863	12.79
**Have any paid work**				
Yes	11,118	88.09	1,716	11.91
No	78,979	88.94	10,019	11.06
Plan to do after completing 9th grade^*^				
Study full-time	11,971	89.81	1,467	10.19
Work full-time	5,844	85.35	972	14.65
Work and study	60,972	89.43	7,458	10.57
Other and don't know	11,296	86.47	1,838	13.53

* *p < 0.01, chi-square test*.

The adjusted models showed that adolescents studying in private schools had a lower prevalence ratio (PR) of hunger (PR:0.985; 95% CI:0.977–0.994), while those from the North (PR:0.983;95% CI:0.974–0.992) and Midwest regions (PR:0.994; 95% CI.983–1.005), women (PR: 1.024; 95% CI: 1.018–1.031), Black (PR: 1.019;95% CI: 1.008–1.031) and indigenous (PR: 1.023;95% CI: 1.005–1.042) adolescent students living in households with more than five people (PR: 1.02; 95% CI: 1.013–1.028), adolescents not living with their father (PR: 1.019; 95% CI: 1.011–1.025), and adolescents planning to work (PR: 1.036; 95% CI: 1.019–1.052) or knowing their plans after completing primary school (PR: 1.025; 95% CI: 1.011–1.04) had a higher prevalence of hunger (Table 2).

**Table 2:** Results of Poisson regression model analysis of the association between self-perceived hunger and sociodemographic variables, Brazil, 2015.

	**Crude Prevalence Ratio**		**CI 95%**	Adjusted Prevalence Ratio^*^		**CI 95%**
**Region**						
North	1			1		
Northeast	0.982^*^	0.972	0.992	0.983^*^	0.974	0.992
Southeast	0.971^*^	0.961	0.982	0.982^*^	0.971	0.992
South	0.978^*^	0.966	0.989	0.989	0.977	1.001
Midwest	0.984	0.973	0.996	0.994	0.983	1.005
**Gender**						
Male	1			1		
Female	1.019^*^	1.013	1.026	1.024^*^	1.018	1.031
**School type**						
Public	1			1		
Private	0.971^*^	0.963	0.979	0.985^*^	0.977	0.994
**Race/Colour**						
White	1			1		
Black	1.029^*^	1.018	1.041	1.019^*^	1.008	1.031
Yellow	1.019	1.000	1.039	1.012	0.994	1.031
Brown	1.009	1.000	1.018	1.001	0.992	1.010
Indigenous	1.031^*^	1.013	1.050	1.023^*^	1.005	1.042
**Age (years)**						
≤13	1			1		
14–15	1.013	1.004	1.022	1.010	1.003	1.021
≥16	1.072^*^	1.057	1.087	1.061^*^	1.047	1.075
**Household size**						
Up to 4 people	1			1		
5 or more people	1.025^*^	1.018	1.033	1.020^*^	1.013	1.028
**Live with the father**						
Yes	1			1		
No	1.022^*^	1.015	1.029	1.019^*^	1.011	1.025
**Plan to do after completing 9th grade**						
Study full-time	1			1		
Work full-time	1.041^*^	1.024	1.057	1.036^*^	1.019	1.052
Work and study	1.003	0.993	1.014	0.999	0.989	1.009
Other and don't know	1.030^*^	1.016	1.045	1.025^*^	1.011	1.040

* Adjusted according to other sociodemographic variables: sex, school type, race, have any paid work, region, age, household size, living with the father, and plans after completing the ninth grade. *p < 0.01.

## Discussion

The results of this study demonstrate that reports of hunger among Brazilian adolescents have exceeded 10%, which is directly related to sociodemographic factors, allowing to determine the most vulnerable populations. To the best of our knowledge, this is the first study using a representative national sample to explore the reports of hunger among this population. The results of this study are consistent with those of previous national research conducted in 2017 and 2018, showing that 7.3% of the population aged 5–17 years experienced hunger (10).

The self-perception of adolescents used to report hunger in this study was shown to be related to measures that represent the scarcity of resources. The variables selected for correspondence analysis concur with those used in other studies (19). FI-driven food changes are accompanied by decreases in the variety of food intake, which can be expressed by consumption of fruits (20, 21). Maternal education has stronger effects than paternal education at the level of income and the cognitive performance of children, and is associated with lower prevalence of childhood undernutrition (22, 23). It is well established that internet access is correlated with income (24) and educational achievement (25).

Therefore, demonstrating the reliability of using a population survey database with a representative sample is essential to assess the prevalence of hunger in a specific population (adolescents). Additionally, the PeNSE is performed at regular intervals (3–4 years); thus, it is possible to determine the status of hunger in this population and, in the future, to access the impact of the pandemic, providing important indicators of the prevalence of hunger and FI in this population.

The data from the PeNSE indicated that reports of hunger were more frequent among public school students than among private school students, similar to the findings of the Brazilian Food Insecurity Scale for public and private school adolescent students in Brazilian capitals (1), reinforcing the importance of the National School Feeding Program (Programa Nacional de Alimentação Escolar-PNAE), which may offer principal daily free meals to many students. However, limited studies have examined the factors associated with the consumption of food offered in school feeding programs. Locatelli et al. (26) observed that consumption of the school lunch offered by the PNAE is influenced by the same determinants, which were also found in this study.

The PNAE is currently designed to contribute to biopsychosocial development, growth, learning, school performance, and formation of healthy food habits through education, food and nutrition activities, and provision of meals, which meet the school nutritional requirements during the scholastic year. Moreover, one of the guidelines of the program is to ensure food and nutrition security (27). Because of the importance of this policy in ensuring food and nutrition security, school meals should be provided during school holidays and vacations, especially for the most vulnerable populations (28).

In terms of race and skin colour, black and indigenous students were the most vulnerable to hunger. A similar result was found in other studies that showed significantly higher prevalence of hunger in households with at least one non-White adolescent (29, 30). The prevalence of hunger was also higher among adolescents who did not live with their fathers, emphasising how difficult it is for women to ensure FNS in their households. Some studies have reported a higher prevalence of moderate-to-severe hunger in households where the head of the family was a woman (31, 32). The PeNSE data also indicated a positive relationship between hunger and female students, demonstrating the greater vulnerability of women even before reaching adulthood, and reinforcing the importance of policies dedicated to them.

Students residing in the North and Midwest regions of the country had the highest prevalence of self-reported hunger. However, in the literature, studies on FI in Brazilian regions have found the highest prevalence of hunger (moderate and severe FI) in the North and Northeast regions because of socioeconomic inequalities that have existed in these regions for years and the absence of public policies that guarantee basic rights to health, food, education, and sanitation (10, 32). A possible explanation for the result in the Midwest region was that the proportion of indigenous students (3.88%) in this area was greater than that in the South (2.26%) and Southeast regions (2.86%), based on the sample evaluated.

Existing evidence shows that the prevalence of hunger is higher among rural households (32, 33); however, the PeNSE only considered the area in which schools were located. Therefore, students living in rural areas may have access to schools in urban areas due to the availability of public-school transportations.

Unlike most studies, this study reflects hunger at the individual level and not at the household level; therefore, we could not find a relationship between FNS and the age of adolescents in the literature. However, the prevalence of hunger was greater among older ninth-grade students (aged ≥ 16 years). This fact may be justified by several vulnerability factors that are associated with hunger, since most students in the ninth grade are aged between 14 and 15 years.

In addition to the relationship between inequality and FNS, which has a direct association with access to food (29), some studies have found the same between FNS and poor nutrition. In Brazil, higher prevalence of underweight has been found among underprivileged children and adolescents. Existing data have also indicated that Black and mixed-race women with low income and low levels of education have higher prevalence of obesity and low stature than more privileged women. In addition, Black and mixed-race individuals are more likely to have low-income status. In contrast, higher-income White men with higher levels of education are more likely to be overweight and obese (34). These data reinforce the need for public policies to promote social equality.

This study has some limitations. PeNSE only contains a single question related to FI and hunger; it asks the students about the frequency of going hungry due to not having sufficient food at home over the past 30 days. This method of assessing hunger could explain the prevalence ratios and CI close to 1, which were significant in multiple analysis due to the large sample size. Although the PeNSE question is insufficient to determine household FI, affirmative responses should be read at the same time as evidence of FI. Additionally, a Brazilian study demonstrated the validity of two-item FI screening to identify families at risk of FI and used a question very similar to that used in this study: “During the past 3 months, has a minor in your household gone hungry and not eaten because there was not enough money to buy food?” (35).

To address the issue of hunger, it is important to evaluate the food system based on five pillars: availability, accessibility, utilisation, stability, and sustainability (36, 37). Hunger is a complex social phenomenon that cannot be hidden through various euphemisms, such as “severe FI” or “very low food security,” and food security will not be achieved using simple methods, such as genomics-based breeding of crops (38).

Childhood and adolescence are directly affected by the impact of austerity policies. Considering the current political situation in Brazil and the dismantling of public policies that fight against hunger and guarantee food and nutrition security, we would like to emphasise the importance of the PNAE and the need for investment in policies that further equality among sexes, races, and permanence in school given the direct relationship between these determinants and reports of hunger.

The COVID-19 pandemic has further aggravated the economic, educational, and public health crisis in Brazil, as the country was one of the most severely affected countries (39) because of the negligence of the federal government. Moreover, public schools remained closed in 2020 and part of 2021, which may have exacerbated hunger and social inequality among students.

## Future Challenges

The results of this study address the self-perception of hunger among Brazilian adolescents. There is a paucity of studies investigating the prevalence of FI and hunger in specific populations, such as adolescents, since most studies have assessed FI in households. Regarding existing policies to help end hunger among adolescents, it is very important to understand the associated factors. Strengthening school feeding programs is essential to tackle FI among students, and the implementation of FI screening in this population is important to identify schools with higher proportions of adolescents experiencing hunger, seeking to increase equity in the PNAE.

Future research can validate this simple tool to evaluate hunger in this specific context, which can be crucial in improving hunger and FI screening. The impact of the COVID-19 pandemic on hunger among students should be further investigated, especially with the closing of schools and reduction of access to food offered in this environment.

## Data Availability Statement

Publicly available datasets were analysed in this study. This data can be found here: https://www.ibge.gov.br/estatisticas/sociais/educacao/9134-pesquisa-nacional-de-saude-do-escolar.html.

## Ethics Statement

The studies involving human participants were reviewed and approved by Brazilian National Commission of Research Ethics–Conep. Written informed consent to participate in this study was provided by the participant's legal guardian/next of kin.

## Author Contributions

DB conceptualised the idea. AA and DB performed the analyses. AA and JR wrote the first draught of the manuscript. HG and DB extensively and critically reviewed the manuscript. All the authors have approved the submission of the manuscript.

## Conflict of Interest

The authors declare that the research was conducted in the absence of any commercial or financial relationships that could be construed as a potential conflict of interest.

## Publisher's Note

All claims expressed in this article are solely those of the authors and do not necessarily represent those of their affiliated organizations, or those of the publisher, the editors and the reviewers. Any product that may be evaluated in this article, or claim that may be made by its manufacturer, is not guaranteed or endorsed by the publisher.
